# Miscellanea

**Published:** 1855-08

**Authors:** 


### MISCELLANEA.

*A Brisk Village in the Medical line.*—Atlanta, Georgia, with a population of a few thousand, is the seat of two Medical schools, and boasts of three Medical journals.

*Professorial Appointments*—Gerge C. Blackman, M. D., has been appointed to the chair of Surgery in the Medical College of Ohio, *vice* Asbury Evans, M. D., resigned.—In the same school Dr. S. G. Armor succeeds Dr. Lawson in the chair of Theory and Practice of Medicine.—Dr. James Graham succeeds Dr. Edwards in the chair of Materia Medica, and Dr. John H. Tate has been elected Professor of Physiology, Hygiene, and Medical Jurisprudence.—Dr. John R. Allen, formerly Professor of Obstetrics in the Medical Department of the Missouri University, has accepted the same chair in the Medical Department of the University of Iowa, at Keokuk.—The vacant chair of Physiology and Pathology in the Rush Medical College has been filled by the appointment of William B. Herrick, M. D.—To the chair of Anatomy, Dr. J. W. Freer has been appointed; to that of Materia Medica, Dr. H. A. Johnson, of Chicago.

In Brooklyn, N. Y., there are 268 Regular Physicians and Surgeons, 35 Homœpathists, 11 Eclectics, 3 Botanics, 3 Female Physicians not classified.

*The Women's Hospital*, in New York, has been publicly inaugurated.

*Jews Hospital*, is the name of a new charity in the city of New York, erected by the munificence and voluntary contributions of members of the Hebrew faith.

*German Medical Society.*—A Medical and Surgical Society of German Physicians has been organized at the University Medical College, New York.

*New Hampshire Medical Journal of Medicine.*—Dr. Charles Bell, of Concord, N. H., has been associated with Dr. G. N. Hubbard, as editor of this journal.

*Aluminium.*—The Emperor of the French has conferred the cross of officer of the Legion of Honor on M. St. Clare Deville, and that of simple member on M. Vohler, for their discovery of the new metal aluminium.

*A Medical Goblet.*—Goblets made of quassia wood are now sold at the leading druggists' shops in New York. Water is poured into them, which, after being left for some minutes, is drank, as a cure for dyspepsia. D. W. Y.

